# Exploring the Father–Adolescent Life Satisfaction Relationship in Light of Fathers’ Personality Traits and Satisfaction with Life: A Transgenerational Perspective

**DOI:** 10.3390/bs15091286

**Published:** 2025-09-20

**Authors:** Tatjana Krstić, Ilija Milovanović, Aleksandra Stojadinović, Željka Nikolašević

**Affiliations:** 1 Department of Psychology, Faculty of Medicine, University of Novi Sad, 21000 Novi Sad, Serbia; zeljka.nikolasevic@mf.uns.ac.rs; 2 Department of Psychology, Faculty of Philosophy, University of Novi Sad, 21000 Novi Sad, Serbia; ilijamilovanovic@ff.uns.ac.rs; 3 Department of Pediatrics, Faculty of Medicine, University of Novi Sad, 21000 Novi Sad, Serbia; aleksandra.stojadinovic@mf.uns.ac.rs

**Keywords:** adolescents, fathers, satisfaction with life, personality traits

## Abstract

This study examines how life satisfaction and personality traits are intergenerationally transmitted from fathers to their adolescent children. The sample comprised 144 father-child dyads (mean child age = 13.65; 55.9% male; mean father age = 43.06). The data were obtained via questionnaires. The Satisfaction With Life Scale served as the measure of life satisfaction. Fathers also completed a brief version of the NEO Personality Inventory. The results showed that fathers’ life satisfaction significantly positively correlated with their children’s life satisfaction, supporting the theory of intergenerational transmission. Unexpectedly, fathers’ high neuroticism positively correlated with adolescents’ life satisfaction, contradicting previous studies suggesting a negative association. Additionally, fathers’ high agreeableness was significantly associated with higher life satisfaction in their children, indicating that agreeable fathers contribute to a supportive and nurturing family environment, enhancing adolescents’ well-being. In conclusion, this study emphasizes how fathers’ personality traits, particularly agreeableness and neuroticism, notably contribute to the psychological and emotional well-being of their adolescent children.

## 1. Introduction

### 1.1. Satisfaction with Life: A Transgenerational Perspective

Over the past several decades, the rise in positive psychology has steered psychological studies towards promoting positive human experiences, optimal human and societal development, and desired characteristics of mental health, which is crucially determined by subjective well-being (Diener et al., 2018b; Wissing, 2022). Subjective well-being is theoretically conceptualized as having two main components: an affective one, referring to the frequency and intensity of pleasant versus unpleasant emotions, and a cognitive one, which relates to an individual’s overall satisfaction with life (Diener et al., 2010). Specifically, the cognitive component reflects how people evaluate their general life satisfaction, independent of specific norms, values, or goals (Pavot & Diener, 1993).

The initial studies of subjective well-being, that is, life satisfaction, focused on the adult population. Due to the importance of this concept for optimal functioning in life at all ages, scientific interest has shifted towards the younger population, including children and adolescents. Recent research supports the view that life satisfaction during childhood and adolescence is a key indicator of psychological development and overall well-being. Beyond its developmental significance, life satisfaction has also been shown to function as a protective factor, mediating the effects of external social relationships on adolescents’ mental health outcomes (Aymerich et al., 2021; Cavioni et al., 2021).

Recognizing the significance of subjective well-being in adults and children, more recent research efforts have centered on behavioral-genetic and family studies (Lucchini et al., 2016; Milovanović et al., 2018; Sadiković et al., 2018). However, findings on the heritability of life satisfaction have not been unambiguous, with estimates of heritability ranging from mere 11% to remarkable 50% (Lucas, 2008). Beyond shared genetic factors, recent studies emphasize the critical role of parental involvement in shaping children’s preferences and attitudes, demonstrating that more engaged parents tend to have children who resemble them more closely in these domains (Zumbuehl et al., 2021). Hence, studies have explored the effects of parental variables on children’s life satisfaction from the perspective of intergenerational transmission, indicating that the effects are stronger in children who are more similar to their parents in the studied aspects (Branje et al., 2020). The parent–child transmission of life satisfaction pertains to evidence of particular traits that have been positively or negative linked to life satisfaction being passed on from parents to their children. Some studies have found intergenerational transmission mechanisms to be responsible for both direct and indirect links between parents’ and children’s subjective well-being (Augustijn, 2022; Clair, 2012; Fan et al., 2023). A strong correlation between the two generations’ life satisfaction levels can persist long after parents and children stop living in the same household (Dobewall et al., 2019). Nonetheless, findings supporting this significant association have not been universally replicated. In one Spanish study, parents had significantly lower subjective well-being compared to their 12–16-year-old children (Casas et al., 2012). In conclusion, despite the steadily growing literature on life satisfaction among children and adolescents, intergenerational transmission of life satisfaction has not yet received sufficient and conclusive empirical support.

Findings in this field can be extremely valuable since it has been shown that life satisfaction is a stable dimension of subjective well-being (Lucas, 2008) and parental influences can have long-term consequences, which is especially challenging when parents transmit their specific disadvantages to their children (Augustijn, 2022). Research findings also indicate that children’s life satisfaction is associated with their fathers’ distress levels in the following year (Powdthavee & Vignoles, 2008). Additionally, a British study showed that children were more consistently affected by paternal versus maternal life satisfaction (Clair, 2012). We consider this significant, especially in countries with lower estimated life satisfaction, such as Serbia. Life satisfaction measures indicate that, on average, respondents from Serbia score one standard deviation lower than respondents from developed countries (Vasić et al., 2011).

### 1.2. Personality Traits and Satisfaction with Life

Since the way people experience their life circumstances greatly depends on their personality traits, it comes as no surprise that studies have consistently confirmed a strong correlation between personality traits and subjective well-being (Anglim et al., 2020; Mõttus et al., 2024b). Researchers have mostly focused on extraversion and neuroticism as two dimensions that explain substantial variance in well-being, with findings indicating that the evaluation of subjective well-being correlates positively with extraversion but negatively with neuroticism (Diener et al., 2003; Das et al., 2020). Fewer studies have examined other basic personality traits and their results have been inconsistent. Some findings have revealed conscientiousness and agreeableness as positive correlates of life satisfaction (Hayes & Joseph, 2003; DeNeve & Cooper, 1998; Soto, 2015).

Conversely, the dimension of openness to experience is not a likely candidate for predicting subjective well-being. The investigation of parental transmission of subjective well-being and personality traits has also mostly focused on extraversion and neuroticism. Studies have found higher subjective well-being in the children of highly extraverted parents, while parents’ high scores on neuroticism have been shown to predict children’s reduced subjective well-being (Fan et al., 2023). Inconsistencies in the significance of all parental personality traits certainly leave room for additional study in this field, particularly from an intergenerational perspective.

### 1.3. The Current Study

Although research has increasingly focused on the intergenerational transmission of life satisfaction, several aspects of this phenomenon remain insufficiently explored. Namely, while previous studies have explored the general role of parental personality in transmitting life satisfaction to children, much of the research has concentrated on mothers, often neglecting the role of fathers (Clair, 2012) despite the undeniable significance of the father’s role during the formative years of childhood, especially in adolescence (Shulman & Seiffge-Krenke, 2015). Furthermore, many studies have not explored the full spectrum of parental personality traits, focusing primarily on dimensions like extraversion and neuroticism, while overlooking the potentially significant roles of others. Thus, this study aimed to expand the existing framework by focusing specifically on fathers’ personality traits and life satisfaction and their influence on adolescents’ life satisfaction.

The decision to focus exclusively on fathers stems from their distinctive role in adolescent socialization, especially within traditional family structures prevalent in many societies, including Serbia. Historically, fathers have primarily been responsible for providing economic support, while mothers have taken on the role of primary caregivers (Ćeriman, 2021). Consequently, fathers have often been excluded from research because of the belief that they are less interested or less involved in parenting, as well as societal perceptions that their main role is to be economic providers (Cabrera et al., 2018). This division of roles suggests that paternal influence on adolescents may differ qualitatively from maternal influence, warranting separate investigation.

Given this dynamic, our study aimed to isolate the distinct influence of fathers on adolescents’ life satisfaction, which may be underexplored in the existing literature. Our study aligns with recent research emphasizing the unique and significant role that fathers play during adolescence. High-quality relationships between fathers and adolescents have been shown to contribute positively to adolescent perseverance, connectedness, and happiness, effects that are distinct from those of maternal or school relationships. This highlights the importance of considering paternal influences separately, as fathers have a crucial impact on adolescents’ subjective well-being, which can foster positive developmental outcomes during adolescence and young adulthood (Walsh et al., 2024). We acknowledge that including data on mothers could provide additional insights into how both parents influence children’s life satisfaction. Future research should explore this avenue and potentially supplement and validate the current study’s results and conclusions.

**Figure 1:**
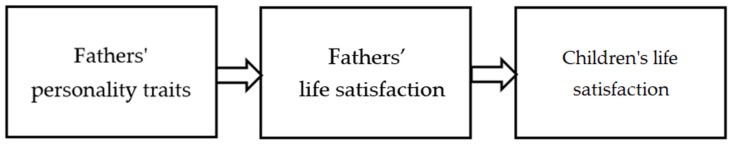
Conceptual model of the current study.

This study specifically sought to explore how fathers’ personality traits and life satisfaction influence adolescents’ life satisfaction. According to the theory of intergenerational transmission, children whose fathers share key personality traits (e.g., extraversion and neuroticism) more commonly inherit their fathers’ life satisfaction, which has long-term effects on their psychological and emotional well-being. Building on previous findings suggesting that life satisfaction is significantly shaped by personality traits (Diener et al., 2003; Fan et al., 2023), we hypothesized that specific personality traits of fathers, such as extraversion and conscientiousness, would positively influence adolescents’ life satisfaction, whereas fathers’ neuroticism would be negatively associated with adolescents’ life satisfaction (Figure 1). Furthermore, we expected that fathers’ life satisfaction would have a stronger predictive effect on adolescents’ life satisfaction than paternal personality traits.

## 2. Materials and Methods

### 2.1. Participants and Procedure

The present study recruited 144 dyads of adolescents (Range_Age_ = 10–15, *M* = 13.65, *SD* = 2.16), and their biological fathers (Range_Age_ = 30–61, *M* = 43.06, *SD* = 5.13). Gender-wise, 55.9% of adolescents were male and 44.1% female. The majority of the fathers were high school graduates (62.3%), married (97.4%), and had two (71.4%) or three (28.6%) children. Data were collected online between 2021 and 2024 (for more information on the data collection procedure, see: Smederevac et al., 2019). They filled out a registration questionnaire requiring them to enter their e-mail address. Subsequently, they were sent a link to a questionnaire comprising all pertinent information about the study and its stages, consent forms, researchers’ contact details, and self-report questionnaires (adolescents and fathers received separate questionnaires and were instructed to respond independently). The study was approved by the Ethics Committee of the University of Novi Sad (approval no. 202010291658_SyRk).

### 2.2. Measures

Paternal personality traits were measured using the NEO-FFI (Costa & McCrae, 1992, 2019), a brief version of the NEO Personality Inventory. Fathers were required to indicate the degree to which they agreed (1 = strongly disagree; 5 = strongly agree) with 60 items referring to the Big Five personality traits: Neuroticism (α = 0.78), Openness (α = 0.70), Agreeableness (α = 0.70), Extraversion (α = 0.71), and Conscientiousness (α = 0.76). The NEO Personality Inventory has been validated for use in Serbian populations (Knežević et al., 2004, the Serbian version). In line with previous studies on personality assessment, we employed the five-factor personality model (Knežević et al., 2004), which has been extensively validated in both normative and clinical samples in Serbia (α > 0.70 for all dimensions).

To measure adolescents’ and fathers’ life satisfaction, we asked them to specify their level of agreement (1 = strongly disagree; 7 = strongly agree) with the five items of the Satisfaction With Life Scale (SWLS), the adult and adolescent version (Diener et al., 1985; the Serbian version for adults and adolescents: Jovanović, 2016, 2019). The SWLS measures the cognitive aspect of satisfaction with life and it showed adequate reliability coefficients in fathers (α = 0.90) and adolescents (α = 0.83) in the actual sample. To ensure the validity of the Satisfaction With Life Scale (SWLS) in this study, we relied on prior research confirming its validity (α > 0.80) and measurement invariance across gender, age, samples, and time in Serbia (Jovanović, 2016, 2019; Jovanović et al., 2020). This validation supports the appropriateness of using the SWLS to make meaningful life satisfaction comparisons across different demographic groups.

### 2.3. Analytic Strategy

Analyses were run in the IBM SPSS 25.0.0.2 software for Windows. The power analysis was conducted using a medium effect size (Cohen’s d = 0.5) for a two-tailed test at a significance level of *p* = 0.05. This analysis showed that a minimum sample of 138 pairs would provide 85% power to detect significant results. The final sample consisted of 144 father–adolescent pairs. Although this sample size allowed us to detect significant results, a larger sample size could provide more stable estimates of correlations. According to Schönbrodt and Perugini (2013), a sample size of at least 260 participants would offer more stable correlation estimates, particularly in simpler research designs.

Data encoding was conducted in the wide format, with every row representing one father–adolescent pair. Pearson’s correlations were calculated for all study variables to determine simple relations between fathers’ characteristics and adolescents’ life satisfaction. A regression analysis was conducted to assess the contribution of fathers’ personality traits to their life satisfaction. Finally, a hierarchical regression analysis served to ascertain how well fathers’ personality traits and life satisfaction predicted adolescents’ life satisfaction. Additionally, the analysis served to identify potential changes in the contribution of personality traits after the introduction of fathers’ life satisfaction in step two.

## 3. Results

Table 1 shows descriptive statistics and bivariate correlations between all study variables. Extraversion, Agreeableness, and Conscientiousness showed positive correlations while Neuroticism correlated negatively with life satisfaction in fathers. Adolescents’ life satisfaction correlated positively with fathers’ Agreeableness and Conscientiousness as well as fathers’ life satisfaction. There were no gender differences in life satisfaction in the group of adolescents, *t*(142) = 1.72, *p* = 0.09. All study variables were normally distributed according to the criterion, −3.00 < *Sk*, *Ku* < 3.00, presented by Tabachnick and Fidell (2021). However, the measure of life satisfaction with respect to the father (SWLS–father), showed a slight negative skew (−1.29). This value still falls within the range considered acceptable for the use of parametric tests. Nevertheless, such skewness may have slightly influenced the strength of associations with other variables, and the results should therefore be interpreted with some caution. Given the negative skew, it is possible that associations with other variables were slightly attenuated due to the limited variance in the lower range. Nevertheless, the skewness remained within acceptable bounds for parametric analyses, and thus the main findings are unlikely to be substantially affected.

A paired samples *t*-test was conducted to compare the mean scores between fathers and adolescents SWLS. Fathers reported significantly higher scores compared to adolescents, t(287) = 14.41, *p* < 0.001. The regression analysis conducted to assess fathers’ personality traits and their life satisfaction showed that these traits accounted for 23% of the variance in fathers’ life satisfaction, F(5, 139) = 7.79, *p* < 0.01. Specifically, Neuroticism negatively contributed to fathers’ life satisfaction, β = −0.22, *p* < 0.05, while Conscientiousness had a positive effect, β = 0.30, *p* < 0.01.

Since fathers’ and adolescents’ life satisfaction levels correlated positively and there were significant links between fathers’ personality traits and their life satisfaction, a hierarchical regression analysis was conducted to discover if fathers’ personality traits had greater explanatory power compared to fathers’ life satisfaction in predicting adolescents’ satisfaction with life. In the first step, we controlled for whether the child lived with the father, as this contextual factor could confound the associations between fathers’ and children’s personality traits and children’s life satisfaction. The analysis also provided insight into potential changes in the contribution of fathers’ personality traits after the introduction of their life satisfaction in step three of the hierarchical regression (Table 2).

The proportion of explained variance in adolescents’ life satisfaction was 11% in the second step and 29% in the third step, after introducing fathers’ life satisfaction into the model, whereby the *F*-change was significant, *p* < 0.01. Fathers’ life satisfaction made the greatest positive contribution to adolescents’ life satisfaction and fathers’ Agreeableness also remained significant in the positive direction, as in the second step of the hierarchical regression. However, in the second step of the analysis, fathers’ Neuroticism gained significance in the positive direction, which suggests the existence of an interaction between fathers’ Neuroticism and fathers’ life satisfaction in the prediction of adolescents’ life satisfaction. Fathers with both higher Neuroticism and greater life satisfaction most likely exhibit behavior that contributes to their children developing greater life satisfaction in adolescence.

## 4. Discussion

The current study points to the existence of the transmission effect of life satisfaction from fathers to their children. This aligns with previous studies reporting significant positive links between parents’ and children’s life satisfaction levels (Augustijn, 2022; Fan et al., 2023). Analyzing individual contributions of predictor variables, we found that fathers’ life satisfaction had a greater predictive value compared to their personality traits when the observed outcome was their children’s life satisfaction.

Although fathers’ neuroticism was not significantly correlated with adolescents’ life satisfaction at the bivariate level, it nevertheless emerged as a significant predictor in the multiple regression model. This discrepancy is most plausibly attributable to a suppressor effect, in which the shared variance with other parental personality traits suppresses irrelevant variance and reveals the unique contribution of neuroticism. Interestingly, the direction of this effect was positive, indicating that higher paternal neuroticism was associated with greater adolescent life satisfaction. Such a finding contrasts with previous research, which has consistently documented negative associations between neuroticism and life satisfaction (Das et al., 2020; Diener et al., 2003). One possible explanation lies in the characteristics of the present sample, where fathers’ average neuroticism scores were relatively low, suggesting limited dysfunctionality. In this light, the observed effect is likely to represent a statistical artifact, as the inclusion of other variables in the model may have masked the true nature of the relationship. This pattern underscores the complexity of the associations under study and highlights the need for cautious interpretation. Replication in independent samples is required before firm conclusions can be drawn regarding the role of paternal neuroticism in adolescents’ subjective well-being.

Given this possibility, some of the explanations we initially proposed warrant reconsideration. One plausible mechanism involves compensatory influences, particularly from mothers. Since mothers are generally more involved in childcare (Ćeriman, 2021; Van Lissa et al., 2019), they or other caregivers might provide additional emotional support and stability, which could help maintain adolescents’ life satisfaction. The peer environment may also play a role. A supportive school climate and positive peer relationships have been shown to foster life satisfaction, especially during adolescence (Roach, 2018). Additionally, children of neurotic fathers may develop resilience and adaptive coping strategies to navigate the challenges of having a neurotic parent (Masten & Barnes, 2018), which can contribute to life satisfaction. Fathers with high neuroticism may be more aware of their emotional difficulties and invest extra effort to maintain positive relationships with their children, leading to quality time and meaningful interactions. Studies have shown no relationship between fathers’ neuroticism and intrusive parenting behavior (Zvara et al., 2019), suggesting that positive father-child interactions may still occur. If adolescents perceive their neurotic fathers as caring, this could positively influence their life satisfaction (Steinberg & Silk, 2002). Furthermore, positive shared experiences, family traditions, and celebrations can also contribute to overall life satisfaction (Diener et al., 2018a). Nevertheless, these interpretations remain speculative, as they are not directly supported by the data in the present study and thus call for further empirical investigation.

Consequently, these findings should be viewed as preliminary and interpreted with caution, pending replication in future research that explores potential statistical artifacts. In summary, while our results offer intriguing insights into the possible association between fathers’ neuroticism and adolescents’ life satisfaction, we recognize that this relationship may reflect a statistical artifact rather than a direct causal effect. Additional studies are essential to validate these findings and to clarify the underlying factors influencing this association.

The results of our study identified the dimension of agreeableness as significant for adolescents’ sense of life satisfaction. The relationship between fathers’ high agreeableness and adolescents’ high satisfaction with life can be explained through various psychological and social mechanisms. In interpersonal relationships, agreeableness is a dimension that includes trust, altruism, empathy, and the need to help others. High agreeableness in a father typically translates to a supportive, nurturing, and harmonious family environment. A positive emotional climate in the family, characterized by closeness and low perceived overcontrol, strengthens the impact of supportive parenting and serves as a protective factor for adolescent well-being (Kapetanovic & Skoog, 2021). Additionally, agreeable fathers are more likely to effectively communicate and aid conflict resolution within the family. This reduces household tension and promotes a harmonious environment (Ravindran et al., 2020). In turn, this leads to agreeable fathers being less likely to create stressful situations and more likely to provide support during stressful times. This buffering effect can help adolescents manage stress more effectively, contributing to their overall satisfaction with life (Leto et al., 2021).

Thus, fathers as well as mothers decisively shape their children’s emotional lives, which has lasting effects on children’s mental health and well-being (Ramakrishnan et al., 2019). In sum, a father’s high agreeableness fosters a supportive, nurturing, and harmonious family environment. This environment enhances adolescents’ emotional security, models positive social behaviors, and provides effective stress-coping mechanisms. These factors collectively contribute to adolescents experiencing higher levels of life satisfaction.

The quality of the father-child relationship likely plays a crucial role in shaping the mechanisms behind the observed associations between paternal personality traits and adolescents’ life satisfaction. Fathers high in agreeableness may create a warm and communicative family atmosphere that strengthens emotional bonds and promotes adolescents’ sense of security and well-being. In addition, the unexpected positive link between paternal neuroticism and adolescent life satisfaction might reflect that some fathers channel their emotional sensitivity into more attentive and responsive caregiving. Such dynamics could foster a close and supportive father-child relationship, which in turn positively influences adolescent outcomes. These findings highlight that not only individual personality traits but also the emotional climate and interaction quality within the father-child dyad are important factors to consider when understanding adolescent psychological development (Palkovitz, 2019).

Our study counterintuitively showed that in addition to fathers’ high agreeableness, high paternal neuroticism had a positive effect on adolescents’ life satisfaction. While these two dimensions seem to be opposites, it is worth considering the possibility that fathers in our study may have manifested their higher neuroticism levels through more sensitive childcare, rather than solely exhibiting negative emotions and creating tension. This idea warrants further investigation into how these two traits might interact and contribute to the family climate and childcare dynamics. Future studies could explore how the interplay between agreeableness and neuroticism in fathers influences adolescents’ life satisfaction, with further empirical support needed to draw definitive conclusions.

Although we initially hypothesized that fathers’ extraversion would positively influence adolescents’ life satisfaction, our results did not support this, which is surprising given the previous literature suggesting such an effect (Das et al., 2020; Sirgy, 2021).

One possible explanation is that while high extraversion in fathers is generally associated with positive social interactions, it may not directly translate into increased life satisfaction among adolescents within the context of this study. Fathers with higher extraversion may be more sociable and open, potentially benefiting their relationship with their children, yet these effects were not evident in our analysis. It is possible that the impact of extraversion operates indirectly, through complex family dynamics and social interactions not captured here. In traditional Serbian families, where fathers often assume the role of economic providers and mothers primarily manage emotional and social interactions, paternal extraversion may have limited influence on adolescents’ emotional well-being. Future research should explore how extraversion affects father–adolescent interactions and whether its impact depends on factors such as relationship quality or family structure.

However, it is important to note that, since our design was based on family correlations, it precluded a clear separation of genetic and shared environmental influences. While we observed familial aggregation of life satisfaction, we could not conclusively determine whether the relationship between fathers and children was solely of genetic origin or influenced by their shared environment. According to previous studies (e.g., Mõttus et al., 2024a), shared environmental factors (such as family, social environment, and life circumstances) can significantly contribute to similarities in life satisfaction among relatives, which our design could not fully clarify.

Given these methodological limitations, future research should employ designs that allow for the separation of genetic and environmental influences, such as genetic studies of twin or adoption samples or studies using multimethod approaches. It would also be valuable to examine the role cross-cultural differences may have in intra-family transmission of life satisfaction, particularly in contexts characterized by significant variances in parental roles and dynamics, including non-traditional families. It is also crucial to continue investigating specific personality traits that may contribute to life satisfaction in order to further clarify their role in the intergenerational transmission of this phenomenon.

Due to this being a cross-sectional study, it was impossible to draw any well-founded conclusions regarding causal relationships between parental variables and children’s life satisfaction. Longitudinal studies would provide a more comprehensive insight into the process and effects of intergenerational transmission over time. Additionally, the small sample size is another limiting factor that should be addressed in future research, which could also purposefully compare different age groups of children. When examining life satisfaction, especially in adolescents, it would be beneficial to include measures that go beyond general life satisfaction, such as specific dimensions of life satisfaction that may be particularly relevant during adolescence. Furthermore, our findings may have limited generalizability due to the study’s cultural context being decisively shaped by the traditional Serbian family structure. Hence, future studies should validate our findings across different cultural settings and family structures and strive to reveal how different parental roles and personal traits affect life satisfaction across diverse sociocultural environments.

Despite these limitations, our study makes several important contributions. It uniquely focuses on the role of fathers in the intergenerational transmission of life satisfaction, an area that has been relatively underexplored in the literature, which has often concentrated on mothers. By investigating a broader range of paternal personality traits alongside life satisfaction, our study expands the existing framework and sheds significant light on the intricate interplay between fathers’ traits and the well-being of their children. In doing so, we call for future research to further investigate the synergistic effects of maternal and paternal influences and their collective impact on adolescents’ emotional and psychological development.

Findings related to intergenerational transmission should be considered in light of their practical implications. Adolescents reporting low life satisfaction could benefit from support programs that strengthen both personal and environmental resources. The results underscore the role fathers play in shaping adolescents’ well-being, particularly through their personality traits and overall life satisfaction. This highlights the need for interventions aimed at supporting fathers, especially those with lower life satisfaction, by helping them understand how their attitudes, emotional well-being, and behavior patterns influence their children.

These insights can be valuable for psychologists, family therapists, and educators working with families, as they suggest that supporting fathers’ well-being and parenting approaches may positively contribute to adolescents’ mental health. Educating fathers about how their personality traits and emotional well-being influence their children could foster a more supportive and nurturing family environment.

## Figures and Tables

**Table 1:** Bivariate Correlation and Descriptives of All Study Variables.

	N	E	O	A	C	SWLS*f*	SWLS*a*
N	-						
E	−0.26 **	-					
O	−0.01	0.10	-				
A	−0.42 **	0.38 **	0.26 **	-			
C	−0.48 **	0.29 **	0.20 **	0.39 **	-		
SWLS*f*	−0.39 **	0.22 **	0.00	0.23 **	0.42 **	-	
SWLS*a*	0.09	0.08	0.15	0.26 **	0.21 **	0.42 **	-
*M*	2.44	3.41	3.07	3.39	3.99	5.34	4.10
*SD*	0.68	0.50	0.63	0.64	0.65	1.16	0.82
*Sk*	0.49	−0.40	0.09	−0.47	−0.74	−1.29	−0.51
*Ku*	0.40	1.60	−0.28	0.01	0.79	2.04	0.04

Note. N—Neuroticism; E—Extraversion; O—Openness; A—Agreeableness; C—Conscientiousness; SWLS—Satisfaction With Life Scale; *f*—father; *a*—adolescent. ** *p* < 0.01.

**Table 2:** Prediction of Adolescents’ Life Satisfaction Based on Father’s Personality Traits and Father’s Life Satisfaction.

	SWLS*a*—Step 1		SWLS*a*—Step 2		SWLS*a*—Step 3	
	*F* = 0.90; *R* = 0.02; *R*^2^ = 0.04		*F* = 2.91 *; *R* = 0.33; *R*^2^ = 0.11; Δ*R*^2^ = 0.07		*F* = 7.99 **; *R* = 0.54; *R*^2^ = 0.29; Δ*R*^2^ = 0.18	
	β	*t*	β	*t*	β	*t*
**LwF**	0.13	1.45	0.03	0.37	0.03	0.36
**N**	-	-	0.05	0.90	0.19	1.98 *
**E**	-	-	−0.06	−0.80	−0.10	−1.13
**O**	-	-	0.07	0.82	0.11	1.29
**A**	-	-	0.21	2.19 *	0.20	2.14 *
**C**	-	-	0.14	1.48	0.02	0.25
**SWLS*f***	-	-	-	-	0.44	5.01 **

Note. LwF—Living with father (1—yes; 0—no). N—Neuroticism; E—Extraversion; O—Openness; A—Agreeableness; C—Conscientiousness; SWLS—Satisfaction With Life Scale; *f*—father; *a*—adolescent. * *p* < 0.05. ** *p* < 0.01.

## Data Availability

The data used in this study is available at https://osf.io/3yq5f/ (accessed on 1 March 2025).

## References

[B1-behavsci-15-01286] Anglim J., Horwood S., Smillie L. D., Marrero R. J., Wood J. K. (2020). Predicting psychological and subjective well-being from personality: A meta-analysis. Psychological Bulletin.

[B2-behavsci-15-01286] Augustijn L. (2022). The intergenerational transmission of life satisfaction between parents and children and the mediating role of supportive parenting. Journal of Family Issues.

[B3-behavsci-15-01286] Aymerich M., Cladellas R., Castelló A., Casas F., Cunill M. (2021). The evolution of life satisfaction throughout childhood and adolescence: Differences in young people’s evaluations according to age and gender. Child Indicators Research.

[B4-behavsci-15-01286] Branje S., Geeraerts S., de Zeeuw E. L., Oerlemans A. M., Koopman-Verhoeff M. E., Schulz S., Nelemans S., Meeus W., Hartman C. A., Hillegers M. H., Oldehinkel A. J., Boomsma D. I. (2020). Intergenerational transmission: Theoretical and methodological issues and an introduction to four Dutch cohorts. Developmental Cognitive Neuroscience.

[B5-behavsci-15-01286] Cabrera N. J., Volling B. L., Barr R. (2018). Fathers are parents, too! Widening the lens on parenting for children’s development. Child Development Perspectives.

[B6-behavsci-15-01286] Casas F., Coenders G., González M., Malo S., Bertran I., Figuer C. (2012). Testing the relationship between parents’ and their children’s subjective well-being. Journal of Happiness Studies.

[B7-behavsci-15-01286] Cavioni V., Grazzani I., Ornaghi V., Agliati A., Pepe A. (2021). Adolescents’ mental health at school: The mediating role of life satisfaction. Frontiers in Psychology.

[B8-behavsci-15-01286] Clair A. (2012). The relationship between parent’s subjective well-being and the life satisfaction of their children in Britain. Child Indicators Research.

[B9-behavsci-15-01286] Costa P. T., McCrae R. R. (1992). Revised NEO personality inventory (NEO-PI-R) and NEO five-factor inventory (NEO-FFI) professional manual.

[B10-behavsci-15-01286] Costa P. T., McCrae R. R. (2019). Srpska standardizacija NEO petofaktorskog inventara NEO-FFI: Forma S [The Serbian standardization of the NEO five-factor inventory NEO-FFI: Form S].

[B11-behavsci-15-01286] Ćeriman J. (2021). Models of Boys’ Gender Socialization in Families in Modern-day Serbia. Etnoantropološki problemi/Issues in Ethnology and Anthropology.

[B12-behavsci-15-01286] Das K. V., Jones-Harrell C., Fan Y., Ramaswami A., Orlove B., Botchwey N. (2020). Understanding subjective well-being: Perspectives from psychology and public health. Public Health Reviews.

[B13-behavsci-15-01286] DeNeve K. M., Cooper H. (1998). The happy personality: A meta-analysis of 137 personality traits and subjective well-being. Psychological Bulletin.

[B14-behavsci-15-01286] Diener E., Emmons R. A., Larsen R. J., Griffin S. (1985). The Satisfaction With Life Scale. Journal of Personality Assessment.

[B15-behavsci-15-01286] Diener E., Kesebir P., Tov W., Leary M. R., Hoyle R. H. (2010). Happiness. Handbook of individual differences in social behavior.

[B16-behavsci-15-01286] Diener E., Lucas R. E., Oishi S. (2018a). Advances and open questions in the science of subjective well-being. Collabra: Psychology.

[B17-behavsci-15-01286] Diener E., Oishi S., Lucas R. E. (2003). Personality, culture, and subjective well-being: Emotional and cognitive evaluations of life. Annual Review of Psychology.

[B18-behavsci-15-01286] Diener E., Oishi S., Tay L. (2018b). Advances in subjective well-being research. Nature Human Behaviour.

[B19-behavsci-15-01286] Dobewall H., Hintsanen M., Savelieva K., Hakulinen C., Merjonen P., Gluschkoff K., Keltikangas-Järvinen L. (2019). Intergenerational transmission of latent satisfaction reflected by satisfaction across multiple life domains: A prospective 32-year follow-up study. Journal of Happiness Studies.

[B20-behavsci-15-01286] Fan H., Li D., Zhou W., Jiao L., Liu S., Zhang L. (2023). Parents’ personality traits and children’s subjective well-being: A chain mediating model. Current Psychology.

[B21-behavsci-15-01286] Hayes N., Joseph S. (2003). Big 5 correlates of three measures of subjective well-being. Personality and Individual Differences.

[B22-behavsci-15-01286] Jovanović V. (2016). The validity of the Satisfaction with Life Scale in adolescents and a comparison with single-item life satisfaction measures: A preliminary study. Quality of Life Research.

[B23-behavsci-15-01286] Jovanović V. (2019). Measurement invariance of the serbian version of the satisfaction with life scale across age, gender, and time. European Journal of Psychological Assessment.

[B24-behavsci-15-01286] Jovanović V., Lazić M., Gavrilov-Jerković V. (2020). Measuring life satisfaction among psychiatric patients: Measurement invariance and validity of the Satisfaction with Life Scale. Clinical Psychology & Psychotherapy.

[B25-behavsci-15-01286] Kapetanovic S., Skoog T. (2021). The role of the family’s emotional climate in the links between parent-adolescent communication and adolescent psychosocial functioning. Research on Child and Adolescent Psychopathology.

[B26-behavsci-15-01286] Knežević G., Džamonja-Ignjatović T., Đurić-Jočić D. (2004). Petofaktorski model ličnosti.

[B27-behavsci-15-01286] Leto I. V., Loginova S. V., Varshal A., Slobodskaya H. R. (2021). Interactions between family environment and personality in the prediction of child life satisfaction. Child Indicators Research.

[B28-behavsci-15-01286] Lucas R. E., Eid M., Larsen J. J. (2008). Personality and subjective well-being. The science of subjective well-being.

[B29-behavsci-15-01286] Lucchini M., Della Bella S., Crivelli L. (2016). Genetic and environmental contributions to life satisfaction. Handbook of research methods and applications in happiness and quality of life.

[B30-behavsci-15-01286] Masten A. S., Barnes A. J. (2018). Resilience in children: Developmental perspectives. Children.

[B31-behavsci-15-01286] Milovanović I., Sadiković S., Kodžopeljić J. (2018). Genetic and environmental factors in emotion regulation and life satisfaction: A twin study. Primenjena Psihologija.

[B32-behavsci-15-01286] Mõttus R., Kandler C., Luciano M., Esko T., Vainik U. (2024a). Familial transmission of personality traits and life satisfaction is much higher than shown in typical single-method studies. PsyArXiv.

[B33-behavsci-15-01286] Mõttus R., Realo A., Allik J., Ausmees L., Henry S., McCrae R. R., Vainik U. (2024b). Most people’s life satisfaction matches their personality traits: True correlations in multitrait, multirater, multisample data. Journal of Personality and Social Psychology.

[B34-behavsci-15-01286] Palkovitz R. (2019). Expanding our focus from father involvement to father–child relationship quality. Journal of Family Theory & Review.

[B35-behavsci-15-01286] Pavot W., Diener E. (1993). Review of the satisfaction with life scale. Psychological Assessment.

[B36-behavsci-15-01286] Powdthavee N., Vignoles A. (2008). Mental health of parents and life satisfaction of children: A within-family analysis of intergenerational transmission of well-being. Social Indicators Research.

[B37-behavsci-15-01286] Ramakrishnan J. L., Garside R. B., Labella M. H., Klimes-Dougan B. (2019). Parent socialization of positive and negative emotions: Implications for emotional functioning, life satisfaction, and distress. Journal of Child and Family Studies.

[B38-behavsci-15-01286] Ravindran N., Hu Y., McElwain N. L., Telzer E. H. (2020). Dynamics of mother–adolescent and father–adolescent autonomy and control during a conflict discussion task. Journal of Family Psychology.

[B39-behavsci-15-01286] Roach A. (2018). Supportive peer relationships and mental health in adolescence: An integrative review. Issues in Mental Health Nursing.

[B40-behavsci-15-01286] Sadiković S., Smederevac S., Mitrović D., Milovanović I. (2018). Behavioral genetics foundations of relations between personality traits and satisfaction with life. Primenjena Psihologija.

[B41-behavsci-15-01286] Schönbrodt F. D., Perugini M. (2013). At what sample size do correlations stabilize?. Journal of Research in Personality.

[B42-behavsci-15-01286] Shulman S., Seiffge-Krenke I. (2015). Fathers and adolescents: Developmental and clinical perspectives.

[B43-behavsci-15-01286] Sirgy M. J. (2021). Effects of personality on wellbeing. The psychology of quality of life: Wellbeing and positive mental health.

[B44-behavsci-15-01286] Smederevac S., Mitrović D., Sadiković S., Milovanović I., Branovački B., Dinić B. M., Nikolašević Ž., Kodžopeljić J., Bugarski Ignjatović V., Semnic M., Vujanić Stankov T., Vučinić N., Oljača M., Pajić D., Stojadinović A., Krstić T., Milutinović A. (2019). Serbian twin registry. Twin Research and Human Genetics.

[B45-behavsci-15-01286] Soto C. J. (2015). Is happiness good for your personality? Concurrent and prospective relations of the big five with subjective well-being. Journal of Personality.

[B46-behavsci-15-01286] Steinberg L., Silk J. S., Bornstein M. H. (2002). Parenting adolescents. Handbook of parenting.

[B47-behavsci-15-01286] Tabachnick B. G., Fidell L. S. (2021). Using multivariate statistics.

[B48-behavsci-15-01286] Van Lissa C. J., Keizer R., Van Lier P. A., Meeus W. H., Branje S. (2019). The role of fathers’ versus mothers’ parenting in emotion-regulation development from mid–late adolescence: Disentangling between-family differences from within-family effects. Developmental Psychology.

[B49-behavsci-15-01286] Vasić A., Šarčević D., Trogrlić A. (2011). Zadovoljstvo životom u Srbiji. Primenjena Psihologija.

[B50-behavsci-15-01286] Walsh C. S., Kliewer W., Sullivan T. N. (2024). Adolescents’ subjective well-being: The unique contribution of fathers. Child & youth care forum.

[B51-behavsci-15-01286] Wissing M. P. (2022). Beyond the “third wave of positive psychology”: Challenges and opportunities for future research. Frontiers in Psychology.

[B52-behavsci-15-01286] Zumbuehl M., Dohmen T., Pfann G. (2021). Parental involvement and the intergenerational transmission of economic preferences, attitudes and personality traits. The Economic Journal.

[B53-behavsci-15-01286] Zvara B. J., Altenburger L. E., Lang S. N., Schoppe-Sullivan S. J. (2019). The role of coparenting in the association between parental neuroticism and harsh intrusive parenting. Journal of Family Psychology.

